# Renally Dosed Cefepime Leading to Cefepime-Induced Neurotoxicity: A Case Report

**DOI:** 10.7759/cureus.52162

**Published:** 2024-01-12

**Authors:** Megan Smith, Chris Mehdizadeh, Avrodet Mourkus, Saad A Ansari

**Affiliations:** 1 Internal Medicine, California University of Science and Medicine, Colton, USA; 2 Internal Medicine, University of California Riverside School of Medicine, Riverside, USA

**Keywords:** cefepime-induced neurotoxicity, altered mental status evaluation, drug-induced seizures, neurology and critical care, renal dosing, pseudomonas endocarditis

## Abstract

Cefepime is a broad-spectrum fourth-generation cephalosporin with activity against both gram-positive and gram-negative bacteria, including *Pseudomonas aeruginosa. *Cefepime is most commonly used for urinary tract infections, soft tissue infections, and febrile neutropenia. Up to 15% of ICU patients on cefepime may experience cefepime-induced neurotoxicity (CIN), with risk factors including renal dysfunction, excessive dosage, elevated serum cefepime concentrations, and history of prior brain injury. The adverse effects of CIN, including encephalopathy, seizures, and coma can be resolved with drug cessation, antiepileptics, or hemodialysis. Here, we present the case of CIN in a 59-year-old female patient with long-term cefepime antibiotic prescription for *Pseudomonas *bacteremia and endocarditis with multiple risk factors for reduced renal function. We discuss the relevant risk factors and preventive measures that may have prevented her from developing CIN, as well as the importance of early recognition and prevention of CIN in patient care.

## Introduction

First described in 1999, cefepime-induced neurotoxicity (CIN) is a well-documented consequence of the use of cefepime in patients with chronic kidney disease, and the rate is much higher in patients with significantly decreased (<30ml/minute) glomerular filtration rates (GFR) [1-3]. The mechanism is believed to be due to cefepime’s ability to cross the blood-brain barrier (BBB), BBB dysfunction, and cefepime’s concentration-dependent gamma-aminobutyric acid (GABA) antagonism [4-6]. CIN symptoms typically manifest around four days after starting cefepime and include encephalopathy, seizures, and EEG changes [7]. The etiology of these symptoms can be difficult to discern in critically ill patients due to varying symptoms that are common to this patient population, and delayed recognition of these drug-induced symptoms may predispose patients to further toxicity [2,8]. Critical illness requiring intensive care and febrile neutropenia were also found to be risk factors for CIN, which may be due to increased BBB permeability in an inflammatory state such as bacteremia [3,8-10].

This article was previously presented as a poster at the American College of Physicians Annual Scientific Meeting for Southern California Chapters I, II, and III on October 7, 2023.

## Case presentation

We present a case of a 59-year-old female patient who presented with a past medical history of systemic lupus erythematosus (SLE), migraine headaches, atrial fibrillation with rapid ventricular response (RVR), intravenous (IV) drug abuse, and prior port-a-cath placement. This patient was admitted at a separate site for evaluation of atrial fibrillation with RVR, anemia, and gastrointestinal (GI) bleeding that required transfusion of four units of packed red blood cells (pRBCs). At this other site, she was found to have *Pseudomonas* bacteremia and infective endocarditis with valve vegetations. Her port-a-cath was removed and the patient was sent home one week into a six-week course of IV cefepime dosed at 2 gm every 24 hours. The patient also had a sputum culture positive for *Pseudomonas aeruginosa* and a urinary tract infection, which grew *Escherichia coli*.

Eleven days into her IV cefepime treatment, paramedics were called by the patient’s neighbors due to new-onset erratic behavior and agitation. Upon arrival at the emergency department, she was noted to be agitated, tachycardia, and hypertensive. Chest X-ray was normal, and CT head without contrast showed nothing acute. Blood urea nitrogen and creatinine were elevated at 34 mg/dL and 2.81 mg/dL, respectively. GFR was 17 ml/minute. Her urine drug screen (UDS) was positive for opiates. All other lab tests were normal including urinalysis, lactate, blood glucose, thyroid-stimulating hormone, and troponin. Cefepime 2 gm every 24 hours was continued while the patient was admitted per her prior antibiotic regimen for *Pseudomonas* coverage. Transesophageal echocardiography (TEE) showed no signs of endocarditis.

**Figure 1:**
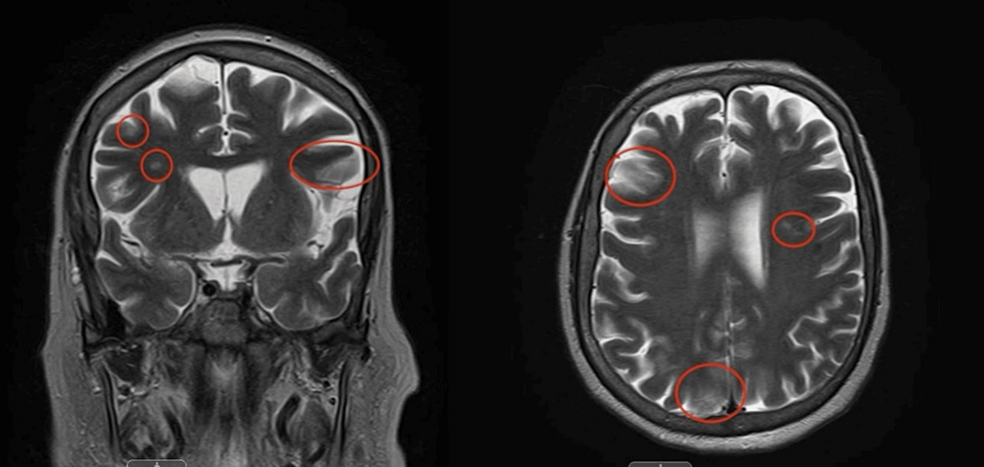
Coronal and Axial T2-weighted MRI showing scatted subcortical and ventricular white matter hyper-intensities. These may represent chronic small vessel ischemic disease or reversible encephalopathy and may be seen after seizures and metabolic disturbances.

On day three of admission and 13 days into IV cefepime treatment, the patient developed non-convulsive status epilepticus, aborted with valproate and lorazepam, after which the patient developed another seizure and was subsequently started on valproate and levetiracetam. After status epilepticus, CIN was proposed as a potential cause, and cefepime was switched to piperacillin-tazobactam. The patient was then admitted to the ICU to intubate and sedate her for burst suppression. A lumbar puncture was performed and cerebrospinal fluid analysis results were all within normal limits. Lupus cerebritis workup was negative. EEG showed general diffuse slowing. Magnetic resonance imaging (MRI) of the head without contrast showed nonspecific findings that may have been indicative of reversible encephalopathy (Figure 1).

Due to worsening renal function, the patient was treated with intermittent hemodialysis. She was also started on pulsatile steroids for five days. Following hemodialysis and steroids, her mental status improved slightly, but she did not return to her baseline mentation. The patient then further deteriorated, eventually coding for 2.5 hours before lifesaving measures were terminated on day 14 of hospital admission, 24 days after long-term cefepime therapy was initiated, having received a total of 13 days of cefepime treatment.

## Discussion

Cefepime has been found to cause neurotoxic symptoms in patients at a median onset of four days of therapy. Symptoms begin with altered mental status (AMS) including somnolence and confusion, which can progress to myoclonus, seizures, status epilepticus, and coma if cefepime therapy is continued. To resolve the neurotoxicity, discontinuation, antiepileptic administration, or hemodialysis can be used with a median symptom resolution of two days [3]. Prior case reports have shown CIN development despite appropriate renal dosing of cefepime as in the current case, and a 2017 systematic review showed that 25% of CIN cases occurred in patients who received proper dosing [3,7]. Several studies have shown complete resolution of symptoms if CIN is recognized and cefepime use is stopped early enough [11].

This patient presented with several underlying conditions that may have predisposed her to the development of AMS including previously diagnosed Pseudomonas bacteremia with endocarditis, known IV drug use with positive urine drug screen for opiates, SLE, and reduced renal function. Determining the etiology of AMS in an ICU setting is often difficult due to the physiological complexity of patients requiring intensive care, but prompt recognition of the underlying cause(s) may improve patient outcomes, as has been demonstrated previously [11].

Although there were many factors complicating this case, the patient was on a renal dose of cefepime (2 gm every 24 hours) appropriate for severe infection with severely reduced GFR (11-30 ml/minute). Per the outside hospital’s records, the patient’s GFR was last noted to be 30 ml/minute prior to long-term cefepime prescription. With this most recent hospital admission, however, the patient’s GFR was further reduced to 17 ml/minute, indicating that she may have suffered some renal insult after discharge from her previous hospital stay that was undetected until the latest admission. Prior to being prescribed cefepime, she was also treated for anemia and a GI bleed, the latter of which may have caused a prerenal acute kidney injury (AKI) and a sharp decline in GFR, although the etiology of her low GFR had not been confirmed. She also had risk factors for intrarenal AKIs including pre-existing SLE that may have impacted her kidney function. Cefepime itself has been published previously as causing acute interstitial nephritis [12]. Although this patient had a severely reduced GFR and presented to the hospital with AMS 11 days into cefepime treatment, she was continued on cefepime for three more days before CIN was recognized as a potential cause of her AMS, after she had already gone into status epilepticus. 

Despite being properly dosed for her severely reduced renal function, the patient still developed symptoms of CIN, EEG changes, nonspecific MRI changes potentially indicative of reversible encephalopathy, and status epilepticus (Figure 1). The etiology of this was likely multifactorial in the current case; infection and inflammation both can affect the integrity of the BBB [10], and our patient had prior diagnoses of SLE, endocarditis, and Pseudomonas bacteremia per outside hospital records. Her kidney function was also acutely lowered with a GFR of 17 ml/minute on presentation in the setting of a recent lower GI bleed requiring four units of pRBCs at an outside hospital, for which her cefepime dosage was correct given her infection severity. Nonetheless, this decreased renal function may have impacted her clearance of cefepime, leading to her increased risk for and development of CIN. Early recognition of the risk of CIN in this patient may have prevented evolution into status epilepticus and may have reversed the encephalopathic symptoms entirely [11]. Due to renal excretion of cefepime, patients with low baseline kidney function or those who are at risk for AKI should be given a different antibiotic altogether and nephrotoxic agents should be avoided in patients receiving cefepime therapy. In the future, if CIN can be ruled out, then attention can be shifted to other possible causes of AMS for patients who are in critical status.

## Conclusions

We want to emphasize the need to improve awareness of CIN, as continued use of cefepime in patients with severely reduced renal function has been shown to cause severe, potentially life-threatening sequelae. As impaired renal function is the biggest risk factor for CIN, patients in need of antibiotics with baseline reduced renal function should have antibiotic susceptibility testing and undergo treatment with agents other than cefepime if available. If cefepime is used in a renally-impaired patient, trough levels can be measured to try and avoid neurotoxic levels above 20 mg/L. However, CIN can occur despite renal dosing having been made and trough levels in patients with CIN can vary. If cefepime is the drug of choice for an infection, patient renal function should be assessed prior to starting antibiotic therapy as well as assessed for risk factors that could lead to reduced GFR, as this puts patients at significant risk for CIN. The differential for AMS is broad with a lot of overlap in symptoms, thus physicians with a high clinical index of suspicion for CIN can make a big difference in patient outcomes. 
